# Evaluation, Management and Therapeutic Approach of Cardiovascular–Kidney–Metabolic Syndrome: A Multidisciplinary Delphi Expert Consensus

**DOI:** 10.3390/jcm14248930

**Published:** 2025-12-17

**Authors:** Domingo Orozco-Beltrán, Borja Quiroga, Alberto Esteban-Fernández, Ana Lorenzo Almorós, Virginia Bellido, Teresa Benedito Pérez de Inestrosa, Rubén de Haro, Xoana Taboada, Juan Carlos Romero-Vigara

**Affiliations:** 1 Department of Clinical Medicine, School of Medicine, Miguel Hernández University, San Juan de Alicante, 03550 Elche, Spain; 2 Service of Nephrology, University Hospital of La Princesa, 28006 Madrid, Spain; borjaqg@gmail.com; 3 RICORS2040 (Belonging to the RICORS [Redes de Investigación Cooperativa Orientada a Resultados en Salud] Program), Instituto de Salud Carlos III, 28029 Madrid, Spain; 4 Service of Cardiology, Severo Ochoa University Hospital, 28914 Leganés, Spain; athalbertus@gmail.com; 5 Facultad de Ciencias de la Salud, Universidad Alfonso X El Sabio, 28691 Villanueva de la Cañada, Spain; 6 Service of Internal Medicine, Gregorio Marañón General University Hospital, Complutense University of Madrid, 28007 Madrid, Spain; alorenzoa@salud.madrid.org; 7 Clinical Management Unit of Endocrinology and Nutrition, Institute of Biomedicine of Sevilla (IBiS), Virgen del Rocío University Hospital, CSIC, University of Sevilla, 41013 Sevilla, Spain; virginiabellido@gmail.com; 8 Médico de Familia, Servicio Andaluz de Salud, 18013 Granada, Spain; teresa.benedito@gmail.com; 9 Medical Affairs Department, Boehringer Ingelheim, 08174 Sant Cugat del Vallés, Spain; ruben.de_haro@boehringer-ingelheim.com; 10 Medical Department, Eli Lilly and Company, 28108 Alcobendas, Spain; taboada_xoana@lilly.com; 11 Health Service of Aragon, 50017 Zaragoza, Spain; jcromerovigara@hotmail.es; 12 IISA—Aragon Health Research Institute, 50009 Zaragoza, Spain

**Keywords:** cardiovascular–kidney–metabolic syndrome, chronic kidney disease, diabetes, metabolic syndrome, obesity, consensus

## Abstract

**Objective:** We aimed to develop multidisciplinary recommendations for the management of cardiovascular–kidney–metabolic (CKM) syndrome in Spain. **Methods:** The Delphi method was used. The final questionnaire comprised 61 statements that were assessed using a 9-point Likert scale of agreement, from 1 = fully disagree to 9 = fully agree. A consensus was reached when 80% of answers in all specialties were in the range of 7–9. The overall median was used as a measure of the strength of agreement. **Results:** A total of 70 (97%) panelists met the selection criteria and completed two rounds, including cardiology (13), endocrinology (12), internal medicine (12), nephrology (14), and primary care (19). Among the 61 statements, a consensus was reached in 54 (89%). The consensus to be highlighted included the following: an excess and/or dysfunction of adipose tissue as the initial driver of CKM syndrome (median 8), CKM syndrome that includes both patients at risk (median 8) and those with existing CVD (median 8), coordination of patient management by the family medicine physician (median 9), the essential role of primary prevention in maintaining CKM health (median 9), the administration of drugs with demonstrated CKM benefit in both early-stage patients (median 9) and those in the advanced stages of the syndrome (median 9), and the importance of lifestyle measures (median 9), with a focus on intensive weight loss (median 9). **Conclusions:** This Delphi consensus offers multidisciplinary recommendations highlighting the importance of early recognition, integrated management, and the implementation of preventive and therapeutic strategies with established cardiorenal and metabolic benefits.

## 1. Introduction

Cardiovascular–kidney–metabolic (CKM) syndrome is defined by the American Heart Association (AHA) Presidential Advisory as “a systemic disorder characterized by pathophysiological interactions among metabolic risk factors, chronic kidney disease (CKD), and the cardiovascular system leading to multiorgan dysfunction and a high rate of adverse cardiovascular outcomes” [1]. This framework emphasizes the interplay of these conditions rather than treating them individually, recognizing shared drivers such as excess adiposity and inflammation. It includes both subjects at risk for cardiovascular disease (CVD) and those with existing CVD [1]. The AHA classifies CKM syndrome into five stages that guide its management [1]: stage 0, absence of CKM risk factors; stage 1, excess and/or dysfunctional adiposity; stage 2, metabolic risk factors and/or moderate- to high-risk CKD; stage 3, subclinical CVD in patients with excess/dysfunctional adiposity, metabolic risk factors, or CKD; and stage 4, clinical CVD in patients with excess/dysfunctional adiposity, metabolic risk factors or CKD.

The burden of CKM syndrome is substantial. In the United States (US), the age-adjusted prevalence in adults is estimated to be 11% for stage 0, 28% for stage 1, 47% for stage 2, 5% for stage 3, and 8% for stage 4. Advanced stages (i.e., stage 3 or 4) are associated with adverse social determinants, such as lower education and income [2,3]. An analysis of time trends in the U.S. revealed an increase in the overall prevalence of CKM syndrome from 1988 to 2018 in both sexes, with greater increases in stage 3 disease among men [4]. The prevalence also increases with age, reaching 50% for advanced stages in individuals aged ≥65 years [5].

Beyond its epidemiological impact, CKM syndrome has major clinical and economic consequences. It is linked to higher health care costs [6,7] and to a marked excess of all-cause and cardiovascular mortality [2,8]. Mortality increases with increasing CKM stage, with a disproportionately high number of deaths associated with advanced stages [2].

Given the complexity of the interplay between the various risk factors and clinical conditions involved in CKM syndrome and their management, there is a growing call to move away from fragmented specialty-based care toward integrated, multidisciplinary models capable of addressing the condition holistically [1,9,10]. While international frameworks, such as those from the AHA, provide a foundation, it is critical to adapt recommendations to the realities of each health care system.

The multidisciplinary consensus presented herein aimed to develop recommendations for the comprehensive evaluation and management of CKM syndrome in Spain.

## 2. Materials and Methods

### 2.1. Study Design

This project was conducted using a modified Delphi method with two rounds. The first Delphi round took place between September and October 2024, and the second round occurred in November 2024. This technique is employed to make recommendations when evidence is limited or contradictory or when there is an overload of information [11]. It has been widely used in health research for various purposes, including clarifying particular issues in health service organizations, defining the role of different professionals in interdisciplinary processes, and developing criteria for the appropriateness of interventions [11,12]. The essential characteristics of the Delphi method are anonymity, iteration, controlled feedback, and statistical aggregation of group responses [11,12], which makes it ideal for addressing emerging and complex topics [12] such as the objective of this project. This study was reviewed and approved by the Ethics Committee of Severo Ochoa University Hospital (Leganés, Spain; Reference 09/24). All health care professionals who participated in the Delphi survey were informed of the nature of the project and agreed to participate by signing a contract.

### 2.2. Selection of Experts

The coordinator of the project selected a group of six experts representing the specialties involved in the project: Cardiology, Endocrinology, Internal Medicine, Nephrology, and Family Medicine. The selection of cardiology, nephrology, and endocrinology was based on the components of the syndrome. We included family and internal medicine because they are cross-sectional medical specialties. Family medicine is especially important because it is usually the gateway to the health system and allows patients to navigate the health care system, including specialist and hospital care coordination and follow-up. Regarding internal medicine, these specialists in adult medicine are specially trained to solve diagnostic problems, manage severe long-term illnesses, and help patients with multiple, complex chronic conditions, such as CRM syndrome. This scientific committee proposed potential experts for screening and to eventually be included in the Delphi, with a target of 12 participants for each specialty involved, except for Family Medicine, which had a target of 20 participants; a greater representation was considered necessary given the key role of family medicine physicians in the early detection and coordination of CKM care.

To be included, participants had to meet the following criteria: have at least 10 years of clinical experience, devote at least 70% of their time to clinical practice, and be active members of a scientific society. They also had to have research experience, as evidenced by at least one CKM syndrome-related publication or communication at a scientific congress in the past five years or accreditation by the Spanish National Commission for the Evaluation of Research Activity and teaching experience.

### 2.3. Development of the Questionnaire

On the basis of the AHA Presidential Advisory on CKM health and a narrative review of the current medical literature, the scientific committee performed and agreed to a questionnaire of 58 statements. They were self-developed by the authors based on their broad knowledge and clinical experience with CRM patients, taking into account the main challenges to be faced, and grouped into three blocks addressing different issues related to the comprehensive management of these patients: (1) evaluation (21 statements): definition, screening, assessments, diagnosis and staging, and health resources; (2) overall management (19 statements): early, comprehensive and interdisciplinary management; role of nurses; and education; and (3) therapeutic approach (18 statements): preventive measures, interventions (lifestyle, pharmacological, and rehabilitation), and follow-up.

### 2.4. Consensus Level

Each recommendation was rated anonymously by the participants using a 9-point Likert scale of agreement, from 1 (“completely disagree”) to 9 (“completely agree”).

A consensus for a specific recommendation/statement was reached when at least 80% of the answers were in the range of 7–9 on the Likert scale. This threshold is within the upper limit of the range commonly used in the literature (50–97%) [13]. To achieve an overall consensus, that threshold had to be reached by each of the specialties involved individually.

The questionnaire was administered via a dedicated website. The results of the first round were analyzed and discussed by the scientific committee. Recommendations/statements with a lack of consensus in any of the specialties involved were assessed by the participants in the second round. Statements that did not achieve consensus in the first round were re-evaluated by the scientific committee to determine whether this was due to the ambiguity of the statement itself, in which case it was reformulated, or because the topic itself was controversial.

### 2.5. Statistical Analysis

Absolute and relative frequencies were used to describe the characteristics of the participants. The final results of the rounds are presented as the percentage of agreement for each specialty (i.e., the proportion of participants who rated the item from 7 to 9 on the Likert scale), the percentage of overall agreement, and the overall median of agreement as a measure of the strength of agreement.

All the data were analyzed using MS Excel Office 365- version 2406.

## 3. Results

**Figure 1:**
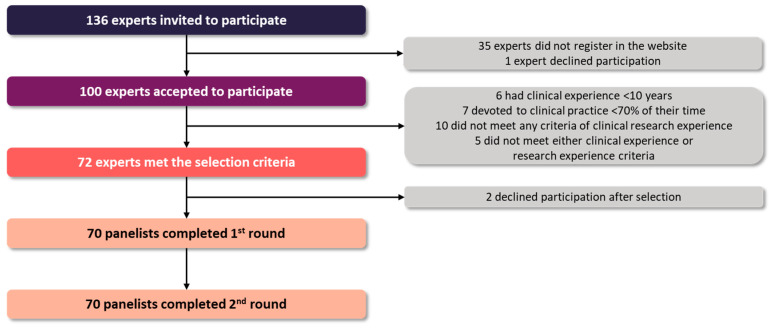
Study flow chart.

A total of 136 experts were invited to participate; 100 (73.5%) accepted the invitation, and 70 (51.5%) met the selection criteria (Figure 1), including 13 cardiologists, 14 nephrologists, 12 endocrinologists, 12 internists, and 19 family physicians. All the participants had prior research experience, with almost half of them having published three or more articles in the past five years (Table S1). Most panelists worked in academic medical centers (87%), had tutored medical residents in the past 5 years (70%), and worked at a highly specialized and complex care hospital (46%) or a community health center (26%).

### 3.1. Overall Results

Seventy panelists (100%) completed both rounds. Among the initial 58 statements, a consensus was not reached for 19 (32.8%), mainly because 15 statements were not agreed upon among the Family Medicine panelists. After reviewing the results of the first round, two statements (items #4 and #13) were split by the scientific committee into five, leading to 22 statements that were evaluated in the second round. In addition, 5 statements were reworded to improve clarity. After two rounds, a consensus was reached in 54 (88.5%) statements.

### 3.2. Evaluation of Patients with CKM Syndrome

A consensus was reached in 20 (83.3%) of the 24 statements in this section (Table 1).

There was a consensus on the definition of CKM syndrome as a complex systemic condition that arises from the multifaceted pathophysiological interactions among metabolic risk factors, CKD, and the cardiovascular system (9), which is pathologically rooted in the excess and/or dysfunction of adipose tissue (8). This concept of CKM syndrome is intended to facilitate the multidisciplinary care process (9) and includes individuals at risk of CVD (8) and those with existing CVD (8).

The experts agreed that screening should be performed in patients with at least one risk factor for any of the CKM conditions (9), and the examinations should include standard physical evaluation (8), key metabolic (including the index for liver fibrosis FIB-4) and renal function parameters (9) and, when clinically indicated, electrocardiography and echocardiography (9). Consensus was also reached for establishing the presence of the cardiovascular, renal, or metabolic conditions of CKM syndrome (see definitions in questions 10 to 12 (Table 1)) and for considering that the presence of one CKM condition should lead to ruling out the other two conditions (9).

There was a consensus that once CKM syndrome is diagnosed, it should be staged according to cardiovascular and renal risks (8). However, the panelists did not reach a consensus on which AHA stages were the appropriate ones (8) because the agreement among Family Medicine physicians was only 79%.

Panelists also reached a consensus on several procedural issues, such as creating a basic analytic profile (9), systematically incorporating these parameters into any analysis (8.5), establishing alerts on the basis of those parameters in electronic health records (9) and coding a diagnosis of CKM in the computer systems (9). The panelists also agreed that the laboratory department (9) and health care managers (9) need to improve the available resources.

There was no consensus about the need for an app aimed at health care professionals (8) or patients (7) to improve the health care of patients with CKM syndrome; none of the specialties reached the threshold of consensus in these two items.

### 3.3. Overall Management of Patients with CKM Syndrome

A consensus was reached for all but one of the 19 statements devoted to overall management (Table 2).

There was consensus on several general measures for its overall management: the need for an early and comprehensive approach (9) aimed at preventing progression and delaying the development of complications (9); the adaptation to clinical scenarios and comorbidities (9) but also to advanced age and the presence of frailty (9); the inclusion of integrated care circuits (9), avoiding consultations with multiple specialists (9) and encouraging multidisciplinary face-to-face consultations; and the provision of a quick answer by referents of each specialty to any referral (9).

With respect to the role of nurses, there was a consensus that they play key roles in anamnesis, physical examination, lifestyle recommendations and patient education (9), as well as in coordination with physicians, in follow-up plans (9). However, a consensus was not reached on their role in CKM screening, largely because of a lack of agreement among Family Medicine physicians (79%). Coordination across specialists involved in the management of CKM syndrome should be led by Family Medicine physicians (9).

There was also a consensus on some procedural issues: the need for quality indicators (9), the usefulness of telemedicine for self-management and communication (9), and the need for a single computer system accessible to all specialists (9).

Finally, the panel underscored the central role of education in CKM management. Educational efforts should extend to the general population by reinforcing primordial prevention strategies (9), to patients by fostering self-care and adherence to therapeutic recommendations (9), and to health care professionals and managers by strengthening knowledge and awareness of the syndrome and its clinical implications (9).

### 3.4. Therapeutic Approach for Patients with CKM Syndrome

The panelists reached a consensus for 16 (88.9%) of the 18 recommendations included in this section (Table 3).

In addition to the clinical situation, the stage of CKM syndrome conditions included the periodicity of monitoring (9), the goals of control (9) and the interventions to be carried out for each condition (9). Primordial prevention was considered essential for maintaining CKM health in individuals without any risk factors (9) and in patients with at least one risk factor associated with CKM syndrome to prevent the occurrence of other risk factors (9).

There was a consensus that, regardless of the overt CKM condition, the approach in patients with CKM syndrome should be comprehensive and intensive (9). The panelists agreed that lifestyle interventions should be implemented in the early stages (9), emphasizing the importance of intensive weight loss at any stage (9). They also agreed that pharmacological management should be based on the corresponding guidelines for each condition (9), emphasizing the use of drugs with beneficial effects on CKM from the early stages (9) to the advanced stages (9); these drugs include sodium–glucose cotransporter-2 inhibitors (SGLT2is), glucagon-like peptide 1 receptor agonists (GLP-1RAs), statins and other lipid-lowering agents, angiotensin-converting enzyme inhibitors (ACEis), angiotensin receptor blockers (ARBs), and mineralocorticoid receptor antagonists (MRAs), among others. There was also a consensus that patients with CKM syndrome and cardiovascular events should be included in comprehensive cardiac rehabilitation programs (9).

There was no consensus on the use of apps for staging and management issues (8) or on telemedicine-related actions on the basis of the stage and psychosocial determinants (8) because they did not reach the consensus threshold by Family Medicine panelists (74% and 79%, respectively). However, panelists agreed on the implementation of telemonitoring for detecting decompensations (8), as well as on computer system functionalities that facilitate the creation of analytic profiles (9) and the definition of monitoring and control objectives (9).

## 4. Discussion

Although CKM syndrome is a new entity involving several specialties, a broad consensus was reached among all panelists, demonstrating the importance of actively addressing this disorder in clinical practice.

The consensus reached on defining CKM syndrome as a complex systemic entity derived from the interplay of three components and initiated by excess and/or dysfunctional adipose tissue should lead physicians to consider multiorganic involvement and the need for early intervention, starting with primordial prevention. The current difficulty in addressing CKM patients holistically is that it has not yet been considered a separate entity; a siloed strategy is used on the basis of pathologies and specialties. This approximation not only undermines an integrative approach but has also been shown to be minimally effective in the early management of individual pathologies, as demonstrated by the low rate of diagnosis of CKD [14]. Establishing CKM syndrome is a first step toward reducing the progression and improving the prognosis in patients with different CKM pathologies. The importance of thoroughly understanding the characteristics of each condition (not just those specific to each specialty) and associated risks will enable an earlier and more holistic approach.

Addressing CKM patients holistically also implies, as agreed upon, the need for multidisciplinary and integrative care. Limited research on the impact of integrated care on these clinical conditions has yielded promising results. An integrated team-based intervention for patients with type 2 diabetes at high risk for cardiovascular disease events conducted at a U.S. university hospital showed an improvement in the use of evidence-based therapies and control of cardiovascular risk factors after one year compared to the status at program entry [15]. Integrated Care Pathways (ICPs) have been developed for chronic diseases, since they are considered beneficial for reducing hospital admissions and re-admissions and for improving adherence to treatment or quality of life in different pathologies [16]. In Spain, a multidisciplinary team has recently developed a new ICP for spondyloarthritis based on Lean Thinking methodology [17]. Its aim was to establish comprehensive care, communication, planning, quality and practice uniformity as pillars of the approach to this disease. After its implementation, this strategy showed reductions in the number of hospital visits and the frequency of visits to hospital pharmacy, better baseline assessment and education, improved treatment and lifestyle modifications adherence. However, ICPs do not exist for CKM syndrome in Spain; an evolved model of ICP could be transferred to the CKM syndrome framework, focusing on making it applicable to the whole range of CKM patients. In our setting, the implementation of heart failure or cardiorenal units has been shown to improve the management of these patients with treatment optimization and a reduction in the use of health resources, such as hospitalizations [18,19].

Within this integrative framework, Family Medicine physicians play a key role in coordination across specialties. They are recognized by all specialties as the point of entry for CKM patients, care coordinators, and follow-up care providers. Early-stage active management of these patients should lead to reduced workloads for all specialties in the medium and long term and, for CKM patients, better quality of life and fewer outcomes. The integration of Family Medicine physicians in multidisciplinary units, currently composed only of in-hospital specialists, would improve the follow-up of advanced CKM patients and their experience as patients with chronic conditions who frequently access health care services. The generation of new CKM units or teams focused on all stages of CKM syndrome and enriched with the expertise of all specialties, including Family Medicine physicians, experts in CKM, and nurses, would slow the progression of these patients, thereby positively affecting the economic expenses of the health system. In our National Health System with virtually universal health coverage [20], all Family Medicine physicians in a health catchment area share the same referral hospital, which facilitates the development of multidisciplinary protocols and clinical pathways. Moreover, the implementation of unique, electronic clinical platforms, shared by primary and hospital care, would improve coordination, physician and patient experience, and patient outcomes.

Health care managers must play a proactive role in the evolution of our system, advocating for optimized health care models that include tools for the early screening of CKM conditions, easy coding of CKM syndrome, and optimization of control objectives, treatment and follow-up of CKM patients. The implementation of basic indicators would offer objective evidence about the effectiveness of the different measures implemented and their potential extrapolation to other hospital departments, regional health care systems or chronic diseases.

Regarding the therapeutic approach, there was very strong agreement on the essential role of preventive interventions throughout the stages of CKM syndrome, from primordial prevention in the first stage to secondary prevention in the advanced stages. The experts agreed that weight loss is crucial in the management of CKM syndrome; however, this is an area with wide room for improvement. Epidemiological surveys have shown that obesity is underrecognized and undertreated in clinical practice [21], even severe obesity [22]. We fully endorse the AHA’s recommendation to enhance the prevention and management of obesity as a clinical and public health priority and a critical area of training for health care professionals involved in the management of these patients [1]. Although not addressed in our consensus, lifestyle interventions should be extended to other factors, such as smoking, which have been associated with the progression of CKM multimorbidity [23]. Furthermore, fostering CKD diagnosis and integrating MASLD are two future action areas to be prioritized by the medical community because of the underestimation of their prevalence and their impact on cardiovascular risk [14,24].

We also agreed that the use of drugs with beneficial effects on CKM syndrome, such as SGLT2is, GLP-1RAs, or MRAs, should be emphasized from the early to the advanced stages of CKM syndrome. These three medicines, together with statins and blockers of the renin–angiotensin system, are considered the pillars of cardiorenal protection [25,26]. Recent publications have highlighted the evidence for relatively new drugs, such as SGLT2is and GLP-1RAs, across CKM syndrome. These molecules play essential roles in mitigating CV risk and metabolic diseases or disturbances, preserving kidney function and reducing liver inflammation, among other benefits [27]. In particular, SGLT2is have multiple pleiotropic effects, including their antioxidant properties, which translate into clinical benefits and support their central role in the treatment of CKM syndrome [28,29]. International and Spanish authors have proposed SGLT2i usage across the CKM spectrum, from stages 1 to 4 of the AHA classification [28,29]. We must consider the current indications of SGLT2is and recognize that in some CKM conditions—or in earlier stages of others—their benefits are hypothetical, limited or only proven in real life.

Several studies have identified the underuse of SGLT2is and GLP-1RAis in clinical practice [30,31], even in patients with diabetes and cardiovascular disease [32]. These findings may reflect a slow uptake of these drugs since more recent studies have shown an increase in their prescription, which seems more marked among cardiologists and nephrologists [33]. In addition, in some clinical contexts, such as in specialized HF units, their use is currently high [34]. Although lack of time, cost, and other factors are associated with the underuse of therapies such as SGLT2is, GLP-1RAs, or MRAs, training specialists in CKM health is essential for overcoming this barrier. The creation of a subspecialty of ‘cardiometabolic medicine’ for internal medicine [35] and a specialty of ‘preventive cardiology’ for cardiologists has been advocated [36]. However, we believe that these initiatives, as isolated measures, maintain the fragmentation of care when seeking integrative management. Interdisciplinary education is needed [37] and should be promoted by scientific societies or other independent scientific organizations or, ideally, included in the curriculum of medical residents of these specialties with specific training on CKM syndrome and its management.

We think that a culture shift towards more intensive use of evidence-based medications and a drive to treat advanced CKM conditions with the sense of urgency they deserve [38], together with the reinforcement of the feeling that each physician seeing a patient is the owner of the process of treatment implementation that must be executed rapidly [39], might favor a slower progression of CKM conditions. A first step towards making this possible could be to leverage SCORE2 [40], SCORE2-OP [41], SCORE2-Diabetes [42], and SCORE2-CKD Add On [43] scores, designed and validated to assess the cardiovascular and renal risks of patients with different CKM conditions, and PREVENT [44] and CKM2S2-BAG [45] scores, which were recently and specifically developed for patients with CKM syndrome, as well as in-hospital treatment initiation and remote and/or algorithmic-based approaches [38].

There was no consensus, overall or in all individual specialties, on the use of apps aimed at health care professionals or patients to improve the management of CKM syndrome. Despite the increasing availability of eHealth tools, their practical implementation in our health care setting is still limited because of the heterogeneity of electronic health records, interoperability issues, and the limited validation of these technologies in longitudinal studies.

Other issues with a lack of consensus, such as the appropriateness of the AHA staging system and the role of nurses in the screening of CKM syndrome, were driven by the results of the Family Medicine panelists. Family Medicine physicians are commonly responsible for a wide range of clinical entities in time-constrained consultations, and they may perceive both the health care apps and the AHA staging system as overly complex or insufficiently easy to handle for their implementation in daily practice. It is also possible that family medicine physicians do not receive adequate training in using these technologies. Incorporating the staging system into information systems would facilitate its use by physicians. In our setting, the role of nurses differs between primary and secondary/tertiary care, is not well-defined and is highly heterogeneous across sites. The extensive development of primary care and community health centers has created a large staff of nurses whose primary focus is health education and preventive activities beyond patient-demand care. In addition, Family Medicine physicians must respond to the demands of patients, which leads to overcrowded consultations [46]. It is necessary to establish preventive objectives in nursing consultations and to evaluate them so that the activity is efficient, as well as to modify the criteria for on-demand care by the physician, also involving nursing in the resolution of patients’ demands for care [47,48]. Overall, these findings highlight the importance of involving primary care professionals in the design and contextual adaptation of CKM care pathways and tools, thereby ensuring their feasibility and relevance in clinical practice.

This publication may serve as a first step toward the evolution of the Spanish health care system, enabling a more comprehensive approach to CKM patients across all medical specialties. Translating these statements into practical, everyday recommendations for all specialists would be a feasible and highly useful action to ensure the implementation, in an easy way, of at least some of these considerations. In addition, CKM initiatives should be promoted in the most favorable health care environments, and these good practices should be shared in common forums, encouraging their extrapolation to other similar realities and advocating for patient equity.

In addition to the inherent features of expert consensus, our study has other limitations. The selection criteria for the panel of experts aimed to choose leading medical professionals who have deep knowledge about CKM syndrome. Additionally, they represent the reality of Spanish doctors, both in their geographical diversity and in their medical specialties or career paths as health care professionals; nevertheless, personal bias of the panelists, inherent to Delphi methodology, must be considered. We do not provide specific recommendations for many of the issues related to the management of each CKM condition because it was beyond the scope of this consensus. Instead, we recommend relying on available guidelines or recommendation documents. However, CKM syndrome is complex, and the assimilation of all guidelines is complex. Therefore, integrated or unified guidelines are needed to facilitate the management of these patients [26,49]. An effort in this direction has been made by a US and European group of multispecialty experts who have assembled a set of detailed recommendations for the comprehensive management of patients with CKM syndrome [50], although its high degree of complexity may make its practical implementation difficult for many physicians. We did not address the essential issue of adverse social determinants of health that, independent of demographic and lifestyle factors, are associated with multimorbidity [51] and mortality [52] in these individuals. Finally, this consensus focuses on applicability within our health care setting, with universal health care coverage and a specific health care organization in mind. While we believe that most recommendations are applicable to other geographical or health care settings, some, particularly those related to organization, may not be applicable or would require adaptation to specific health care contexts.

We believe that this consensus has two important strengths, namely, its multidisciplinary nature and the fact that we have tried to offer a practical framework to guide clinicians in the management of CKM syndrome, transitioning from a conceptual perspective to a clinically operable entity, and thus providing specific recommendations.

In conclusion, diagnosing and treating CKM syndrome remains a clinical challenge. This Delphi consensus offers multidisciplinary recommendations highlighting the importance of early recognition, integrated management, and the implementation of preventive and therapeutic strategies with established cardiorenal and metabolic benefits. These statements complement existing guidelines by providing expert consensus that may inform more coordinated models of care. Furthermore, they help medical managers, clinical experts and other decision-makers in being more innovative and launching new programs to address CKM conditions in a more comprehensive way. Taken together, these findings represent important first steps toward mitigating the burden of CKM syndrome and promoting more integrated approaches to care. The growing understanding of the connections between these major organ systems and the rapidly evolving evidence supporting the adoption of the CKM syndrome, its integrative management, the benefits of recommended treatments for CKM conditions, and the implementation of optimized care models mandate periodic updates of this consensus.

## Figures and Tables

**Table 1:** Evaluation of patients with cardio–kidney–metabolic syndrome.

Statement/Recommendation	Round	CAR	END	FM	IM	NEPH	Global	Median	Result
01. The concept of CKM syndrome is intended to facilitate the multidisciplinary care process of patients with a clinical management approach based on the cardiovascular–renal–metabolic continuum.	2	100%	100%	89%	92%	100%	96%	9	CONSENSUS
02. CKM syndrome is defined as a complex systemic entity resulting from the multidirectional pathophysiological interaction between metabolic risk factors, CKD and the cardiovascular system, which multiply the risks of the development and progression of each condition, as well as an increased risk of cardiovascular and renal events.	1	100%	100%	84%	92%	93%	93%	9	CONSENSUS
03. The basis of CKM syndrome focuses on excess and/or dysfunction of adipose tissue, which leads to a pro-inflammatory, pro-oxidative, and insulin-resistant state that accelerates metabolic damage and increases the risk of cardiovascular and kidney disease.	2	100%	100%	84%	92%	93%	93%	8	CONSENSUS
04a. CKM syndrome includes both people at risk for CVD due to the presence of metabolic risk factors, CKD, or both, as well as people with existing CVD potentially related to metabolic risk factors or CKD.	2	100%	100%	89%	92%	79%	91%	9	LACK OF CONSENSUS
04b. CKM syndrome includes individuals at risk for CVD due to the presence of metabolic risk factors, CKD, or both.	2	92%	100%	84%	83%	93%	90%	8	CONSENSUS
04c. CKM syndrome includes people with existing CVD potentially related to metabolic risk factors or CKD, or both.	2	100%	100%	89%	92%	86%	93%	8	CONSENSUS
05. Screening for CKM syndrome should be performed for patients with at least one risk factor associated with any of the CKM conditions and structured based on each of the CKM conditions, regardless of the specialist who performs it.	2	100%	100%	89%	100%	93%	96%	9	CONSENSUS
06. Physical evaluation should include measurement of blood pressure, body mass index, abdominal circumference, cardiopulmonary auscultation, and detection of peripheral edema.	1	100%	100%	95%	83%	86%	93%	8	CONSENSUS
07. Basic laboratory tests should include blood glucose, hemoglobin A1c, lipid profile, glomerular filtration rate (eGFR), urine albumin, and FIB-4.	2	100%	92%	89%	83%	100%	93%	9	CONSENSUS
08. Depending on the clinical context, electrocardiogram and, if available, echocardioscopy should be performed to evaluate cardiac function and detect arrhythmias or ventricular hypertrophy.	2	100%	92%	95%	92%	100%	96%	9	CONSENSUS
09. In patients with at least one of the CKM conditions, the presence of the other two conditions should be proactively analyzed.	1	100%	100%	95%	100%	100%	99%	9	CONSENSUS
10. The cardiovascular condition of CKM syndrome is present when subclinical conditions or clinical cardiovascular events related to atherosclerotic cardiovascular disease, coronary heart disease, heart failure, or atrial fibrillation are determined.	1	100%	92%	89%	100%	86%	93%	8	CONSENSUS
11. The renal condition of CKM syndrome is present when eGFR < 60 mL/min/1.73 m^2^ or albumin/creatinine ratio > 30 mg/g is/are maintained for at least 3 months.	1	100%	100%	84%	100%	100%	96%	9	CONSENSUS
12. The metabolic condition of CKM syndrome is present when overweight/obesity, abdominal obesity, and/or dysfunctional adipose tissue (manifested as prediabetes) are diagnosed, with or without the presence of other metabolic risk factors (hypertriglyceridemia, high blood pressure, metabolic syndrome, or diabetes).	1	92%	83%	84%	100%	93%	90%	8	CONSENSUS
13a. An app aimed at health care professionals would favor the diagnosis, treatment and monitoring of CKM patients.	2	77%	50%	79%	75%	79%	73%	8	LACK OF CONSENSUS
13b. An app aimed at patients would favor the diagnosis, treatment and monitoring of CKM patients.	2	38%	50%	58%	58%	50%	51%	7	LACK OF CONSENSUS
14. Patients with CKM should be classified into different stages according to their cardiovascular–renal risk to facilitate their management by health care professionals.	1	100%	100%	89%	92%	100%	96%	8	CONSENSUS
15. Stages 0–4 * established by the AHA presidential advisory are appropriate for patients with CKM. * 0, no cardiometabolic risk factors; 1, excessive or dysfunctional adiposity; 2, metabolic risk factors and/or CKD; 3, subclinical CVD in CKM; 4, clinical CVD in CKM.	2	92%	100%	79%	92%	93%	90%	8	LACK OF CONSENSUS
16. Creating a basic analytical profile of CKM syndrome would help diagnose patients with risk factors.	1	100%	92%	84%	92%	100%	93%	9	CONSENSUS
17. The key parameters for diagnosing CKM syndrome should be systematically included in any analysis and be updated in computer systems with appropriate periodicity.	1	100%	92%	84%	92%	93%	91%	8,5	CONSENSUS
18. It should be encouraged that, in the presence of anomalies in the basic analytical parameters, the electronic health record should generate alerts to identify CKM patients.	1	100%	100%	89%	92%	93%	94%	9	CONSENSUS
19. It should be encouraged that computer systems should allow coding of a diagnosis of CKM syndrome.	1	100%	100%	84%	100%	93%	94%	9	CONSENSUS
20. Laboratory services should be involved in improving the available resources in terms of computer systems and requests for tests.	1	100%	100%	95%	92%	93%	96%	9	CONSENSUS
21. Health care managers must be involved in improving the available resources to allow an optimal care model for patients with CKM syndrome.	1	100%	100%	95%	92%	100%	97%	9	CONSENSUS

AHA, American Heart Association; CAR, Cardiology; CKD, chronic kidney disease; CKM, cardio–kidney–metabolic; CVD, cardiovascular disease; eGFR, estimated glomerular filtration rate; END, Endocrinology; FM, Family Medicine; IM, Internal Medicine; NEPH, Nephrology.

**Table 2:** Overall management of patients with cardio–kidney–metabolic syndrome.

Statement/Recommendation	Round	CAR	END	PC	IM	NEPH	Global	Median	Result
22. Priority should be given to an early and comprehensive approach from the specialty that receives the patient to improve the quality of care regardless of the reason for consultation or admission.	1	100%	100%	95%	100%	86%	96%	9	CONSENSUS
23. The aim of any intervention is to prevent the progression and delay the development of complications from the onset of CKM syndrome.	1	100%	100%	95%	100%	93%	97%	9	CONSENSUS
24. The management of CKM patients is conditioned by the different clinical scenarios and comorbidities they present.	1	92%	100%	95%	100%	93%	96%	9	CONSENSUS
25. Elderly or frail CKM patients require their management to be adapted to their situation.	1	100%	100%	95%	100%	93%	97%	9	CONSENSUS
26. Integrated care circuits must be established with multidisciplinary reference teams that establish efficient and agile communication channels.	2	100%	100%	89%	83%	100%	94%	9	CONSENSUS
27. Optimal patient care must be provided, reducing their passage through several specialists to those situations in which it is beneficial for the patient, based on preestablished criteria (advanced stages, instability, etc.)	1	100%	92%	95%	100%	100%	97%	9	CONSENSUS
28. Multidisciplinary face-to-face consultations reinforce the optimal approach for patients with CKM.	2	100%	83%	89%	83%	86%	89%	9	CONSENSUS
29. The referents of each specialty must respond quickly to the interconsultations from other specialties, and the queries of other doctors from their own specialty.	1	100%	100%	89%	83%	100%	94%	9	CONSENSUS
30. Basic quality indicators must be established to assess the results of the implemented measures objectively.	2	100%	100%	95%	100%	93%	97%	9	CONSENSUS
31. Nurses should play an active role in screening for CKM syndrome.	2	100%	100%	79%	100%	100%	94%	9	LACK OF CONSENSUS
32. The role of the nursing staff is key in terms of anamnesis, basic physical examination, hygienic–dietetic–sanitary recommendations, and education of the patient with CKM syndrome.	1	85%	83%	95%	92%	86%	89%	9	CONSENSUS
33. Nurses should play an active role in the follow-up plans of patients with CKM in coordination with physicians.	1	92%	100%	89%	100%	93%	94%	9	CONSENSUS
34. Family physicians must play a leading role in the coordination among specialties in the management of patients with CKM.	1	92%	100%	89%	83%	86%	90%	9	CONSENSUS
35. Telemedicine should help to improve patient engagement in self-management of the disease and in communication with the health care professional.	2	100%	92%	84%	100%	86%	91%	9	CONSENSUS
36. Clinical records in a single computer system easily consulted and accessible by all specialists must be ensured.	1	100%	100%	95%	100%	100%	99%	9	CONSENSUS
37. Education of the general population regarding the primordial prevention * of risk factors associated with the onset of CKM syndrome should be reinforced. * “Primordial prevention” refers to preventing the occurrence of risk factors before they develop. According to the American Heart Association (AHA), primordial prevention includes creating and maintaining conditions that minimize the occurrence of disease risk factors, as part of a broader approach to reducing the burden of cardiovascular disease. (“Value of primordial and primary prevention for cardiovascular disease: a policy statement from the American Heart Association” W. Weintraub, S. Daniels, L. Burke + 8 more · 23 Aug 2011).	1	100%	92%	84%	100%	86%	91%	9	CONSENSUS
38. Patient health education should be reinforced so that they understand CKM syndrome and its inherent risks, as well as to empower them in their self-care and to comply with the recommendations from the health professionals.	1	100%	100%	95%	100%	100%	99%	9	CONSENSUS
39. Training of all health professionals on CKM syndrome and its associated risks should be encouraged.	1	100%	100%	95%	100%	93%	97%	9	CONSENSUS
40. Managers’ knowledge of the CKM syndrome and its implications should be reinforced.	1	100%	100%	89%	100%	100%	97%	9	CONSENSUS

CAR, Cardiology; CKM, cardio–kidney–metabolic; END, Endocrinology; FM, Family Medicine; IM, Internal Medicine; NEPH, Nephrology.

**Table 3:** Therapeutic approach for patients with cardio–kidney–metabolic syndrome.

Statement/Recommendation	Round	CAR	END	PC	IM	NEPH	Global	Median	Result
41. Preventive measures should be adopted for patients with at least one risk factor associated with any of the CKM conditions.	1	100%	100%	89%	100%	93%	96%	9	CONSENSUS
42. The diagnosis of CKM syndrome should involve continuous follow-up with a stipulated periodicity depending on the stage and clinical situation of the patient to evaluate the evolution and adjust treatment.	1	100%	100%	89%	100%	100%	97%	9	CONSENSUS
43. Control goals for each CKM condition according to stage and clinical situations should be established.	1	100%	100%	100%	100%	100%	100%	9	CONSENSUS
44. The interventions to be carried out for each CKM condition should be established according to the stage of the CKM syndrome and clinical situations.	1	100%	100%	95%	100%	100%	99%	9	CONSENSUS
45. In subjects without any CKM risk factors, cardiovascular–renal–metabolic health maintenance measures should be implemented from an early age aimed at primordial prevention *. * “Primordial prevention” refers to preventing the occurrence of risk factors before they develop. According to the American Heart Association (AHA), primordial prevention includes the creation and maintenance of conditions that minimize the occurrence of disease risk factors, as part of a broader approach to reducing the burden of cardiovascular disease (“Value of primordial and primary prevention for cardiovascular disease: a policy statement from the American Heart Association” W. Weintraub, S. Daniels, L. Burke + 8 more · 23 Aug 2011)	1	92%	100%	84%	92%	93%	91%	9	CONSENSUS
46. In patients with at least one risk factor associated with CKM syndrome *, primordial prevention of other risk factors associated with it should be carried out. * Alterations in glucose metabolism, alterations in kidney function; sleep disorders; gestational diabetes; early menopause; adverse effects on pregnancy; mental health or psychosocial problems; drug, alcohol or tobacco use; sedentary lifestyle or poor eating habits.	1	100%	100%	95%	100%	100%	99%	9	CONSENSUS
47. A comprehensive and intensive approach should be adopted in patients with CKM, regardless of the overt CKM condition(s).	2	100%	100%	95%	92%	93%	96%	9	CONSENSUS
48. The pharmacological approach to each CKM condition must be carried out based on the corresponding guidelines, protocols, or recommendations related to each of them.	1	100%	100%	95%	100%	93%	97%	9	CONSENSUS
49. Lifestyle measures should be implemented from the early stages of CKM syndrome.	1	100%	100%	95%	92%	100%	97%	9	CONSENSUS
50. Intensive weight loss should be emphasized at any stage of CKM syndrome.	2	100%	100%	95%	92%	86%	94%	9	CONSENSUS
51. Drugs with demonstrated cardiovascular, renal, and/or metabolic benefits * should be administered from the early stages of CKM syndrome, within their authorized indications. * Drugs such as SGLT2i, GLP-1RA, statins and other lipid-lowering agents, ACEi/ARB, MRA, etc.	1	100%	83%	95%	83%	93%	91%	9	CONSENSUS
52. Drugs with demonstrated cardiovascular, renal, and/or metabolic benefits * should be administered even when patients are in advanced stages of CKM syndrome, according to the established objectives, within their authorized indications. * Drugs such as SGLT2i, GLP-1AR, statins and other lipid-lowering agents, ACEi/ARB, MRA, etc.	1	85%	92%	95%	100%	93%	93%	9	CONSENSUS
53. All patients with CKM and a cardiovascular event should be included in a comprehensive cardiac rehabilitation program tailored to their needs.	2	100%	100%	89%	92%	86%	93%	9	CONSENSUS
54. A validated app would facilitate staging of patients with CKM, assessing their cardiovascular–renal risk, updating their control objectives, and proposing measures to be implemented.	2	92%	83%	74%	83%	100%	86%	8	LACK OF CONSENSUS
55. Actions related to telemedicine should be recommended according to the stage of the patients with CKM and their psychosocial conditions.	2	100%	92%	79%	100%	100%	93%	8	LACK OF CONSENSUS
56. Telemonitoring should be implemented to detect decompensations early as well as avoid hospitalizations and consultations.	2	92%	100%	84%	100%	100%	94%	8	CONSENSUS
57. The creation of an analytical profile of CKM syndrome would help to better assess the risk of these patients throughout follow-up.	1	100%	100%	84%	92%	100%	94%	9	CONSENSUS
58. Computer systems should alert on the measures to be implemented to help achieve the control and monitoring objectives.	1	100%	100%	84%	100%	93%	94%	9	CONSENSUS

ACEi, angiotensin-converting enzyme inhibitor; ARB, angiotensin receptor blocker; CAR, Cardiology; CKM, cardio–kidney–metabolic; END, Endocrinology; FM, Family Medicine; GLP-1RA, glucagon-like peptide 1 receptor agonist; IM, Internal Medicine; MRA, mineralocorticoid receptor antagonist; NEPH, Nephrology; SGLT2i, sodium–glucose cotransporter-2 inhibitor.

## Data Availability

All the data pertaining to this project are presented in the manuscript.

## Supplementary Materials

The following supporting information can be downloaded at https://www.mdpi.com/article/10.3390/jcm14248930/s1, Table S1: professional characteristics of the panelists.
