# Supplementary material for: Evaluation, Management and Therapeutic Approach of Cardiovascular–Kidney–Metabolic Syndrome: A Multidisciplinary Delphi Expert Consensus
Source: J Clin Med. 2025 Dec 17;14(24):8930. doi: 10.3390/jcm14248930 (PMC12734211; doi:10.3390/jcm14248930)
Supplement: Supplementary file 1 [file jcm-14-08930-s001.zip › jcm-4002499-supplementary.pdf]

Supplementary Table S1. Professional characteristics of the panelists.

	N=70
**Research experience (last 5 years)**	
Published articles	
0	9 (12.9%)
1	7 (10.0%)
2	20 (28.6%)
≥3	34 (48.5%)
Congresses and communications	
1	5 (7.1%)
2	18 (25.7%)
≥3	47 (67.2%)
Six-year research period accreditation	5 (7.1%)
**Teaching experience**	
Working at an academic medical center	61 (87.1%)
Tutoring medical residents within the last 5 years	49 (70.0%)
Two or more lectures at congresses or meetings within the last 5 years	67 (95.7%)
Coordination of a course for a scientific society within the last 5 years	43 (61.4%)
**Medical center**	
Community health center	18 (25.7%)
1st level hospital	5 (7.1%)
2nd level hospital	15 (21.4%)
3rd level hospital	32 (45.8%)
